# Detection of the pancreas-specific gene in the peripheral blood of patients with pancreatic carcinoma

**DOI:** 10.1038/sj.bjc.6690699

**Published:** 1999-09

**Authors:** T Kuroki, T Tomioka, Y Tajima, K Inoue, Y Ikematsu, K Ichinose, J Furui, T Kanematsu

**Affiliations:** Department of Surgery II, Nagasaki University School of Medicine, 1-7-1 Sakamoto, Nagasaki, 8528201, Japan

**Keywords:** pancreatic carcinoma, pancreas-specific gene, reverse transcription polymerase chain reaction, peripheral blood

## Abstract

The prognosis of patients with pancreatic carcinoma remains very poor. To improve the therapeutic results, the early detection of this cancer is needed. The present study was performed to detect the pancreas-specific gene, chymotrypsinogen, in the peripheral blood from patients with pancreatic carcinoma by using reverse transcription polymerase chain reaction (RT-PCR) in order to evaluate the clinical significance of this gene. Ten patients with pancreatic carcinoma, two with acute pancreatitis, three with chronic pancreatitis and ten control subjects were examined for the presence of chymotrypsinogen using RT-PCR techniques in the peripheral blood. To confirm that the chymotrypsinogen gene was expressed in a pancreas-specific manner, the expression of chymotrypsinogen in various types of human adult tissue was evaluated by RT-PCR. The specific band of the chymotrypsinogen gene was detected in the pancreas. Serial dilution studies demonstrated the chymotrypsinogen gene to be detected at a concentration of one pancreatic cell per 10^6^ peripheral blood cells. Seven out of the ten (70%) patients with pancreatic carcinoma were found to be positive based on the RT-PCR findings. In contrast, no pancreas-specific gene was detected in the peripheral blood of any patients with acute pancreatitis, chronic pancreatitis or the control subjects. Our observations show that the detection of the pancreatic specific gene, chymotrypsinogen, is therefore useful as a genetic diagnostic marker in pancreatic carcinoma. © 1999 Cancer Research Campaign


*British Journal of Cancer* (1999) **81**(2), 350–353
© 1999 Cancer Research Campaign
Article no. bjoc.1999.0699

### Detection of the pancreas-specific gene in the peripheral blood of patients with pancreatic carcinoma

**T Kuroki, T Tomioka, Y Tajima, K Inoue, Y Ikematsu, K Ichinose, J Furui and T Kanematsu**

Department of Surgery II, Nagasaki University School of Medicine, 1-7-1 Sakamoto, Nagasaki 8528201, Japan

**Summary** The prognosis of patients with pancreatic carcinoma remains very poor. To improve the therapeutic results, the early detection of this cancer is needed. The present study was performed to detect the pancreas-specific gene, chymotrypsinogen, in the peripheral blood from patients with pancreatic carcinoma by using reverse transcription polymerase chain reaction (RT-PCR) in order to evaluate the clinical significance of this gene. Ten patients with pancreatic carcinoma, two with acute pancreatitis, three with chronic pancreatitis and ten control subjects were examined for the presence of chymotrypsinogen using RT-PCR techniques in the peripheral blood. To confirm that the chymotrypsinogen gene was expressed in a pancreas-specific manner, the expression of chymotrypsinogen in various types of human adult tissue was evaluated by RT-PCR. The specific band of the chymotrypsinogen gene was detected in the pancreas. Serial dilution studies demonstrated the chymotrypsinogen gene to be detected at a concentration of one pancreatic cell per $10^6$ peripheral blood cells. Seven out of the ten (70%) patients with pancreatic carcinoma were found to be positive based on the RT-PCR findings. In contrast, no pancreas-specific gene was detected in the peripheral blood of any patients with acute pancreatitis, chronic pancreatitis or the control subjects. Our observations show that the detection of the pancreatic specific gene, chymotrypsinogen, is therefore useful as a genetic diagnostic marker in pancreatic carcinoma.

© 1999 Cancer Research Campaign

**Keywords:** pancreatic carcinoma; pancreas-specific gene; reverse transcription polymerase chain reaction; peripheral blood

Pancreatic carcinoma is a major cause of human cancer death. In Japan, it is currently the fifth leading cause of cancer-related mortality (Ministry of Health and Welfare, 1992). Pancreatic carcinoma is difficult to diagnose, and a definitive curative resection is possible in only approximately 10% of all patients (Japanese Cancer Society, 1995). Therefore, if pancreatic carcinomas can be detected at an early stage, then the surgical results should improve.

In recent years, an extremely sensitive polymerase chain reaction (PCR) technique has been developed for the diagnosis of various cancers (Harker et al, 1995; Mori et al, 1995; Eltahir et al, 1998). In pancreatic carcinoma, one of the most useful molecular markers of this genetic method is K-*ras* oncogene (Motojima et al, 1991; Tada et al, 1991). Many researchers have reported that approximately 90% of all pancreatic carcinomas contained a K-*ras* oncogene activated by a mutation at codon 12 (Smith et al, 1988; Almoguera et al, 1998; Bos, 1998). This particular genetic alteration thus showed the K-*ras* oncogene to be a sensitive marker for the presence of pancreatic carcinoma, and the K-*ras* mutation is also easily detectable.

Circulating tumour cells have been reported to be detected in the peripheral blood using the PCR technique (Tada et al, 1993; Nomoto et al, 1996). In neuroblastoma, Mattano et al (1992) described a highly sensitive detection assay for circulating neuroblasts in the peripheral blood using reverse transcription PCR (RT-PCR), with the tissue-specific gene as a tumour marker. We thus consider the pancreas-specific gene as a tumour marker to be useful in diagnosing pancreatic carcinoma. In the present study, we focused our attention on the chymotrypsinogen gene, which is a pancreas-specific gene.

*Received 8 September 1998*
*Revised 8 April 1999*
*Accepted 12 April 1999*

*Correspondence to:* T Kuroki

### PATIENTS AND METHODS

### Patients

The subjects in this study comprised ten patients with pancreatic carcinoma (six male, four female; median age 61 years, range 48–77 years), two with acute pancreatitis (two male; 49 and 58 years) and three with chronic pancreatitis (two male, one female; 38, 47 and 68 years), who were all treated at our clinic. Ten healthy volunteers were also included as controls (six male, four female; median age 42 years, range 30–49 years).

### RNA extraction

We collected the peripheral blood before operation in seven patients with pancreatic carcinoma, while the other patient blood samples were collected at the time of diagnosis. The peripheral blood (10 ml) samples from the subjects were taken in the presence of potassium ethylenediaminetetraacetic acid (EDTA) and then were promptly transported to the laboratory at 4°C for immediate processing. In preparing and handling the RNA, we took great care to avoid any degradation by contamination with RNAases. The total cellular RNA was extracted from peripheral blood using Trizol (Life Technologies, Inc., Grand Island, NY, USA). The integrity of the RNA was checked electrophoretically and quantified spectrophotometrically.

**Table 1** Profile of the pancreatic cancer patients and the results of chymotrypsinogen RT-PCR

| Patient no. | Age/Sex | Tumur size (cm)[a] | Confirmation of malignancy | Stage[c] | RT-PCR assay[d] |
|---|---|---|---|---|---|
| 1 | 75/F | 3.5 | Operation | I | + |
| 2 | 48/M | 4.0 | Biopsy | IV | + |
| 3 | 50/M | 3.0 | Operation | III | + |
| 4 | 77/F | 4.0 | Operation | III | – |
| 5 | 69/M | 4.0 | Combined methods[b] | – | + |
| 6 | 52/F | 3.0 | Operation | III | – |
| 7 | 71/F | 4.0 | Operation | III | – |
| 8 | 47/M | 5.0 | Operation | IV | + |
| 9 | 52/M | 4.5 | Operation | I | + |
| 10 | 66/M | 5.5 | Combined methods | – | + |

[a]Tumour size at its greatest dimension. [b]Including clinical examination, radiologic imaging and follow-up. [c]According to the 1987 UICC TNM classification of malignant tumours. –: Stage is unknown. [d]+: positive for chymotrypsinogen gene. –: negative for chymotrypsinogen gene.

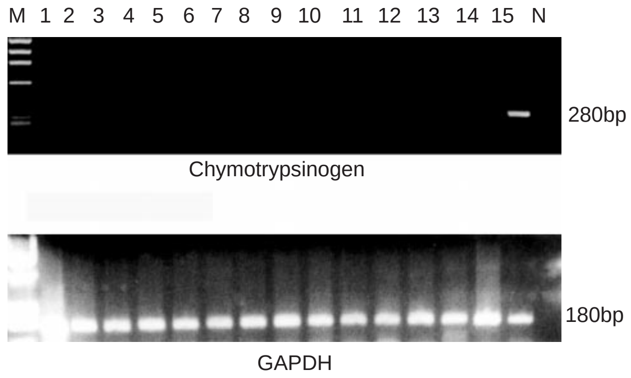

**Figure 1** Specificity of the chymotrypsinogen RT-PCR assay. Chymotrypsinogen and GAPDH total RNA expression in various types of normal tissue was analysed by nested RT-PCR, and the results of 2% agarose gel electrophoresis containing 0.5 mg ml$^{-1}$ ethidium bromide are shown. Lane M, size marker (f X174/Hae III digest); lanes 1–14, various tissue specimens (bile duct, gall bladder, skin, fat, muscle, peripheral blood, ureter, small intestine, urinary bladder, prostate, colon, stomach, papilla of Vater, liver); lane 15, pancreas; lane N, negative control (no template RNA included)

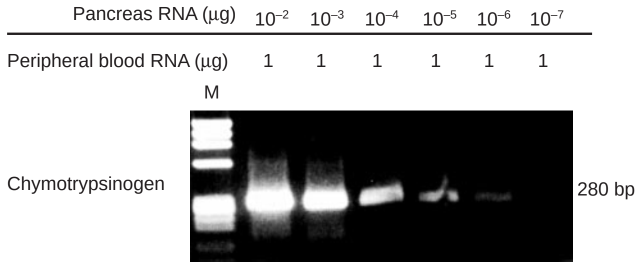

**Figure 2** Sensitivity of the chymotrypsinogen RT-PCR assay. Detection sensitivity was determined by performing serial dilutions of total RNA from pancreatic tissue and preparing the mixture with total RNA from the peripheral blood. Lane M, a size marker (f X174/Hae III digest)

### RT-PCR

First-strand cDNA was synthesized using SuperScript II reverse transcriptase (Gibco-BRL Life Technologies, Inc., Gaithersburg, MD, USA) and random hexanucleotide primers (Pharmacia Inc., Uppsala, Sweden). Four milligrams of total RNA were incubated with 100 ng of random hexanucleotide primers in 11 µl of solution for 5 min at 65°C. After chilling on ice, 4 ml of fivefold synthesis buffer (250 mM Tris–HCl, pH 8.3, 375 mM potassium chloride, 15 mM magnesium chloride), 2 µl of 100 mM dithiothreitol, 1 µl of dNTP (2.5 mM concentrations each of dATP, dCTP, dGTP and dTTP), 1 µl of RNAase inhibitor (40 units µl$^{-1}$; Toyobo, Tokyo, Japan) and 1 µl of SuperScript II reverse transcriptase (200 units µl$^{-1}$) were added. The reaction mixture was then incubated for 1 h at 37°C and the reaction was terminated by heating at 85°C for 10 min and stored at –20°C until use. To standardize the samples and confirm the presence of a cDNA template in each sample, a housekeeping gene, glyceraldehyde-3-phosphate dehydrogenase (GAPDH), was also co-amplified. Chymotrypsinogen gene-specific oligonucleotide primers synthesized for the nested RT-PCR analysis, the sequences of the four primers used were: outer sense, 5′-CTCATCAGCGAGGACTGG-3′; outer anti-sense, 5′-CAGGGCTGCCTGCTGCAG-3′; inner sense, 5′-CCCACTGCGGGGTCAGGA-3′; inner antisense, 5′-GGGGTCTTGTTGGCGTTGTA-3′.

The first-round PCR was carried out in a reaction mixture (25 µl) containing 10 mM Tris (pH 8.3), 50 mM potassium chloride, 2.5 mM magnesium chloride, 200 mM dNTP, 1.0 unit of *Taq* polymerase (Takara Shuzo Co. Ltd, Tokyo, Japan), 100 pmol primers A and B, and 1 µl of template cDNA. The samples were amplified at 35 PCR cycles in the first step for 30 s at 94°C, 30 s at 55°C and 30 s at 72°C, with a final extension step at 72°C for 5 min. One millilitre of each reaction product was then transferred into second tubes containing the identical reaction mixture except for the primers, C and D. These second step PCR cycles were identical to the first step PCR. Ten microlitres of the second-step PCR products were then electrophoresed on 2% agarose gel, followed by ethidium bromide staining. The fragment size amplified from the GAPDH total RNA templates by RT-PCR was 180 base pair (bp), and the nested RT-PCR fragment size from the chymotrypsinogen total RNA was 280 bp. ‘No RT’ controls for all RT-PCR reactions were run as above except that water was substituted for reverse transcriptase.

### RESULTS

### Specificity and sensitivity of chymotrypsinogen RT-PCR

To confirm the chymotrypsinogen gene expressed in a pancreas-specific manner, we examined the expression of the chymotrypsinogen gene in various types of human adult tissue,

*© 1999 Cancer Research Campaign* 

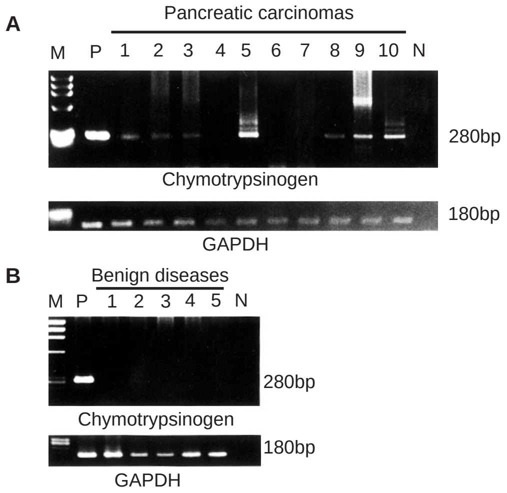

**Figure 3** Detection of chymotrypsinogen RNA from the peripheral blood of patient samples. (**A**) Lanes 1–10 correspond to patients 1–10 (Table 1); lane M, size marker (f X174/Hae III digest); Lane P, positive control (human pancreas tissue RNA); lane N, negative control (no template RNA included). (**B**) Lanes 1 and 2, acute pancreatitis; lanes 3–5, chronic pancreatitis including the bile duct, gall bladder, skin, fat, muscle, peripheral blood, ureter, small intestine, urinary bladder, prostate, colon, stomach, papilla of Vater, liver and pancreas by RT-PCR. As shown in Figure 1, RT-PCR for chymotrypsinogen revealed a band of a 280 bp fragment specifically in the pancreas.

Sensitivity was determined by performing serial dilutions of total RNA extracted from normal pancreatic tissue, and by preparing mixtures with total RNA extracted from normal peripheral blood to represent $10^{-2}$–$10^{-7}$ μg RNA of normal pancreatic tissue in 1 mg RNA of normal peripheral blood. The chymotrypsinogen gene was detected at a concentration of $10^{-6}$ μg normal pancreatic tissue RNA in 1 μg normal peripheral blood RNA (Figure 2).

### Patient samples

The RT-PCR assay revealed the peripheral blood from the ten healthy volunteers used as control subjects, to be negative for the chymotrypsinogen gene (data not shown). This assay resulted in seven positives from the peripheral blood specimens from the ten patients with pancreatic cancer (Figure 3), while tumour tissue specimens were found to be positive for this gene in all cancer patients (data not shown). However, no chymotrypsinogen-specific band was detected in the peripheral blood from two patients with acute pancreatitis and three patients with chronic pancreatitis (Figure 3).

The association between the chymotrypsinogen RT-PCR analysis and the clinical data of pancreatic carcinoma patients is summarized in Table 1. Among the ten patients analysed, a histological confirmation was obtained from surgical specimens in seven patients and from biopsy specimens in one patient. In two patients, the diagnosis of pancreatic carcinoma was made based on the physical stigmata and the findings of a radiological examination. These two patients did not undergo an operation, because widespread lymph node metastases were detected using a computerized tomography scan. The histopathological examination revealed adenocarcinoma in seven patients and mucinous cyst adenocarcinoma in one patient (patient no. 6). Two patients were classified to be at UICC stage I, one was at stage III, two were stage IV in the positive cases according to the findings of the RT-PCR assay. All three patients were also stage III in the cases diagnosed as negative with the RT-PCR assay.

### DISCUSSION

Chymotrypsinogen is an inactive precursor of chymotrypsin, and this enzyme is synthesized and secreted by the pancreas (Tomita et al, 1989). The human chymotrypsinogen gene was confirmed to be localized on chromosome 16q23 by fluorescence in situ hybridization and G-band analysis (Hou et al, 1993). In our experiments, the chymotrypsinogen gene was detected specifically in the pancreatic tissue among the 15 normal tissue specimens including the peripheral blood. These results indicate that when circulating pancreatic cancer cells are found in the peripheral blood, a diagnosis of pancreatic carcinoma can be made. We hypothesized that an extremely sensitive assay had the ability to detect a small number of circulating cancer cells in the peripheral blood. In fact, Tada et al (1993) reported circulating pancreatic cancer cells in the peripheral blood in two of six patients using the PCR technique to any detect *ras* gene mutations. In the present study, detection sensitivity studies using a serial dilution test revealed that a chymotrypsinogen RT-PCR assay is sufficiently sensitive to detect one pancreatic cell in $10^6$ peripheral blood cells, provided that the yield of total RNA from a pancreatic cell is equal to that from a peripheral blood cell. This sensitivity is more sensitive than that reported in the detection of micrometastases by either a cytokeratin 19 (Noguchi et al, 1996) or CEA RT-PCR (Nakanishi et al, 1997) assay using the RT-PCR technique.

The present study demonstrated that the chymotrypsinogen gene could be detected in the peripheral blood based on the RT-PCR findings in seven of the ten patients with pancreatic carcinoma, but not in the ten healthy volunteers, two patients with acute pancreatitis or the three patients with chronic pancreatitis. We therefore considered the chymotrypsinogen gene in the peripheral blood to be a specific tumour marker of pancreatic carcinoma.

The presence of K-*ras* mutations in various clinical samples was thus found to be useful especially in confirming the diagnosis of pancreatic carcinoma. However, several reports also demonstrated K-*ras* gene mutations to be found in hyperplasia associated with chronic pancreatitis (Yanagisawa et al, 1993; Tada et al, 1996). Furthermore, this mutation of the K-*ras* gene has also been reported in various human carcinomas (Bos et al, 1987; Anwar et al, 1993; Kuo et al, 1994). On the other hand, cytokeratin 19 has been reported to be a specific and sensitive molecular marker for the detection of micrometastasis in various cancers (Schoenfeld et al, 1994; Noguchi et al, 1996). We do not consider this marker to be a pancreatic carcinoma-specific marker, because cytokeratin 19 has been reported to be a specific epithelial cell marker (Datta et al, 1994). We need to find the specific molecular marker of pancreatic carcinoma. The results presented in this study suggest that our chymotrypsinogen RT-PCR method is useful for specifically detecting the presence of pancreatic carcinoma cells among the thousands of normal cells.

To confirm the diagnosis of pancreatic carcinoma, various clinical samples were used, including needle biopsy specimens (Shibata et al, 1990), pancreatic juice (Kondo et al, 1994), duodenal fluid (Wilentz et al, 1998) and bile (Ajiki et al, 1995) from a choledochal drainage tube. However, the collection of sufficient samples for a molecular diagnosis is often difficult.

 © 1999 Cancer Research Campaign

However, the collection of peripheral blood is rapid, easy and safe. We therefore studied the possibility of making a genetic diagnosis of pancreatic carcinoma by using peripheral blood.

The establishment of an easy method that can detect pancreatic carcinomas at an earlier stage, when they are still resectable, may thus help to improve the patient outcome. In the present study, the pancreatic carcinoma tumour size did not show any significant difference regarding the positivity of the chymotrypsinogen RT-PCR assay (positive average 4.2 cm versus negative average 3.7 cm). Two stage I cases were found to show a positive result based on the RT-PCR assay findings. These results suggested that this easy and sensitive assay may be a valuable diagnostic tool in the clinical diagnosis of pancreatic carcinoma at an early stage using peripheral blood specimens. However, the negative cases for the RT-PCR assay were all stage III. The reason we could not detect the positive results of this RT-PCR assay in three cases of advanced pancreatic carcinoma remains unknown. We still need to prospectively study this chymotrypsinogen RT-PCR assay using peripheral blood specimens of patients with pancreatic carcinoma. We believe that the diagnostic role for this assay must be further verified through the analysis of more patients and for longer periods.

In conclusion, we demonstrated the chymotrypsinogen RT-PCR assay to be a sensitive, simple and specific diagnostic method for identifying pancreatic carcinoma at an early stage. This diagnostic method using genetic techniques may potentially become a routine diagnostic laboratory procedure and may thus contribute to an improved outcome of patients with pancreatic carcinoma.

### ACKNOWLEDGEMENT

We thank Mr Brian Quinn for his critical comments on our manuscript.

### REFERENCES

Ajiki A, Onoyama H, Yamamoto M, Fujimori T, Maeda S and Saitoh Y (1995) Detection of point mutations in K-ras gene at codon 12 in bile from percutaneous transhepatic choledochal drainage tubes for diagnosis of biliary strictures. *Int J Pancreatol* **18**: 215–220

Almoguera C, Shibata D, Forrester K, Matin J, Arnheim N and Perucho M (1988) Most human carcinomas of the exorine pancreas contain mutant c-K-ras genes. *Cell* **53**: 549–554

Anwar K, Nakakuki K, Naiki H and Inuzuka M (1993) ras gene mutations and HPV infection are common in human laryngeal carcinoma. *Int J Cancer* **53**: 22–28

Bos JL (1998) The ras gene family and human carcinogenesis. *Mutat Res* **195**: 255–271

Bos JL, Fearson ER, Hamilton SR, Verlaan-de Vries M, van Boom JH, van der Eb AJ and Vogelstein B (1987) Prevalence of ras gene mutations in human colorectal cancers. *Nature* **327**: 293–297

Datta YH, Adams PT, Drobyski WR, Ethier SP, Terry VH and Roth MS (1994) Sensitive detection of occult breast cancer by the reverse transcriptase polymerase chain reaction. *J Clin Oncol* **12**: 475–482

Eltahir EM, Mallinson DS, Birnie GD, Hagan C, George WD and Purushotham AD (1998) Putative marker for the detection of breast carcinoma cells in blood. *Br J Cancer* **77**: 1203–1207

Harker R, Glantz MJ, Glantz L, Lekos A, Sorenson GD, Honsiger C and Levy NB (1995) A comparison of polymerase chain reaction examination of cerebrospinal fluid and conventional cytology in the diagnosis of lymphomatous meningitis. *Cancer* **77**: 543–548

Hou DX, Ozawa K, Tomita N, Maeda Y, Hashiguchi T, Yokoyama K and Soeda E (1993) Genomic cloning and partial characterization of human chymotrypsinogen gene. *Jpn J Human Genet* **38**: 371–380

Japanese Cancer Society and The Pancreatic Cancer Registration Committee of the Japan Pancreas Society (1995) The present study of pancreatic cancer registration. *Gann Monograph on Cancer Research*. No 43: 107–117

Kondo H, Sugano K, Fukuyama N, Kyogoku A, Nose H, Shimada K, Ohkura H, Ohtsu A, Yoshida S and Shimosato Y (1994) Detection of point mutations in the K-ras oncogene at codon 12 in pure pancreatic juice for diagnosis of pancreatic carcinoma. *Cancer* **73**: 1589–1594

Kuo MYP, Jeng JH, Chiang CP and Hahn LJ (1994) Mutations of Ki-ras oncogene codon 12 in betel quid-chewing-related human oral squamous cell carcinoma in Taiwan. *J Oral Pathol Med* **23**: 70–74

Mattano LA, Moss TJ and Emerson SG (1992) Sensitive detection of rare circulating neuroblastoma cells by the reverse transcriptase-polymerase chain reaction. *Cancer Res* **52**: 4701–4705

Ministry of Health and Welfare, Statistics and Information Department, Minister's Secretariat (1992) Vital Statistics of Japan. pp. 272–87. Tokyo

Mori M, Mimori K, Inoue H, Barnard GF, Tsuji K, Nanbara S, Ueno H and Akiyoshi T (1995) Detection of cancer micrometastasis in lymph nodes by reverse transcriptase-polymerase chain reaction. *Cancer Res* **55**: 3417–3420

Motojima K, Tsunoda T, Kanematsu T, Nagata Y, Urano T and Shuku H (1991) Distinguishing pancreatic carcinoma from other periampullary carcinomas by analysis of mutation in the Kirsten-ras oncogene. *Ann Surg* **214**: 657–662

Nakanishi H, Kodera Y, Torii A, Hirai T, Yamamura Y, Kato T, Kito T and Tatematsu M (1997) Detection of carcinoembryonic antigen-expressing free tumor cells in peritoneal washes from patients with gastric carcinoma by polymerase chain reaction. *Jpn J Cancer Res* **88**: 678–692

Noguchi S, Hiratsuka M, Furukawa H, Aihara T, Kasugai K, Tamura S, Imaoka S, Koyama H and Iwanaga T (1996) Detection of gastric cancer micrometastases in lymph nodes by amplification of keratin 19 mRNA with reverse transcriptase-polymerase chain reaction. *Jpn J Cancer Res* **87**: 650–654

Nomoto S, Nakao A, Kasai Y, Harada A, Nonami T and Takagi H (1996) Detection of ras gene mutations in perioperative peripheral blood with pancreatic adenocarcinoma. *Jpn J Cancer Res* **87**: 793–797

Schoenfeld Y, Luqmani D, Smith S, O'Reilly S, Shousha S, Sinnett HD and Coombes RC (1994) Detection of breast cancer micrometastases in axillary lymph nodes by using polymerase chain reaction. *Cancer Res* **54**: 2986–2990

Shibata D, Almoguera C, Forrester K, Dunitz J, Martin SE, Cosgrove MM, Perucho M and Arnheim N (1990) Detection of c-K-ras gene codon 12 mutations in fine needle aspirates from human pancreatic adenocarcinomas. *Cancer Res* **50**: 1279–1283

Smith VTHB, Boot AJ, Smits AMM, Fleuren GJ, Cornelisse CJ and Bos JL (1988) K-ras codon 12 mutations occur very frequently in pancreatic adenocarcinoma. *Nucleic Acids Res* **16**: 7773–7782

Tada M, Omata M and Ohta M (1991) Clinical application of ras gene mutation for diagnosis of pancreatic adenocarcinoma. *Gastroenterology* **100**: 233–238

Tada M, Omata M, Kawai S, Saisho H, Ohta M, Saiki RK and Sninsky JJ (1993) Detection of ras gene mutations in pancreatic juice and peripheral blood of patients with pancreatic adenocarcinoma. *Cancer Res* **53**: 2472–2474

Tada M, Ohashi M, Shiratori Y, Okudaira T, Komatsu Y, Kawabe T, Yoshida H, Machinami R, Kishi K and Omata M (1996) Analysis of K-ras gene mutation in hyperplastic duct cells of the pancreas without pancreatic disease. *Gastroenterology* **110**: 227–231

Tomita N, Izumoto Y, Hrii A, Doi S, Yokouchi H, Ogawa M, Mori T and Matsubara K (1989) Molecular cloning and nucleotide sequence of human pancreatic prechymotrypsinogen cDNA. *Biochem Biophys Res Commun* **158**: 569–575

Wilentz RB, Chung CH, Sturm PDJ, Musler A, Sohn TA, Offerhaus GJA, Yeo CJ, Hruban RH and Slebos RJC (1998) K-ras mutations in the duodenal fluid of patients with pancreatic carcinoma. *Cancer* **82**: 96–103

Yanagisawa A, Ohtake K, Ohhashi K, Hori M, Kitagawa T, Sugano H and Kato Y (1996) Frequent c-Ki-ras oncogene activation in mucous cell hyperplasias of pancreas suffering from chronic inflammation. *Cancer Res* **53**: 953–956

*© 1999 Cancer Research Campaign*
